# Development of a flat jet delivery system for soft X-ray spectroscopy at MAX IV

**DOI:** 10.1107/S1600577524006611

**Published:** 2024-08-22

**Authors:** Tamires Gallo, Luigi Adriano, Michael Heymann, Agnieszka Wrona, Noelle Walsh, Gunnar Öhrwall, Flavia Callefo, Slawomir Skruszewicz, Mahesh Namboodiri, Ricardo Marinho, Joachim Schulz, Joana Valerio

**Affiliations:** a Lund University Sweden; b MAX IV Laboratory, Sweden; c European XFEL Germany; d Universität Stuttgart Germany; f Deutsches Elektronen Synchrotron (DESY) Germany; e Brazilian Synchrotron Light Laboratory, LNLS Brazilian Center for Research in Energy and Materials (CPNEM) Brazil; g Institute of Physics, Brasilia University (UnB), 70.919-970Brasília, Brazil; h Institute of Physics Federal University of Bahia 40.170-115Salvador Brazil; Bhabha Atomic Research Centre, India

**Keywords:** liquid jet spectroscopy, 3D-printed flat jet nozzle, photoelectron spectroscopy, photoelectron angular distribution, soft X-ray absorption spectroscopy

## Abstract

One of the most challenging aspects of X-ray research is the delivery of liquid sample flows into the soft X-ray beam. Currently, cylindrical microjets are the most commonly used sample injection systems for soft X-ray liquid spectroscopy. However, they suffer from several drawbacks, such as complicated geometry due to their curved surface. In this study, we propose a novel 3D-printed nozzle design by introducing microscopic flat sheet jets that provide micrometre-thick liquid sheets with high stability, intending to make this technology more widely available to users. Our research is a collaboration between the EuXFEL and MAX IV research facilities. This collaboration aims to develop and refine a 3D-printed flat sheet nozzle design and a versatile jetting platform that is compatible with multiple endstations and measurement techniques. Our flat sheet jet platform improves the stability of the jet and increases its surface area, enabling more precise scanning and differential measurements in X-ray absorption, scattering, and imaging applications. Here, we demonstrate the performance of this new arrangement for a flat sheet jet setup with X-ray photoelectron spectroscopy, photoelectron angular distribution, and soft X-ray absorption spectroscopy experiments performed at the photoemission end­station of the FlexPES beamline at MAX IV Laboratory in Lund, Sweden.

## Abstract 2

Our work presents recent advancements in 3D-printed nozzles for liquid jet spectroscopy, made possible by the availability of nozzle development and testing facilities at EuXFEL, Germany. We also demonstrate the performance of one of these custom-designed nozzles in a new flat jet system under commissioning at MAX IV Laboratory in Lund, Sweden, introducing a new injection method to analyze flat liquid surfaces and opening new possibilities for the spectroscopy community.

## 1. Introduction

X-ray scattering and spectroscopy experiments are widely used in various fields of science. However, their success often relies on the stability of the sample delivery system. Over the last three decades, cylindrical liquid jets have been widely used as sample injection systems in experimental setups that aim to study samples in solution and suspension. Cylindrical liquid jets, however, have several limitations that make their operation at beamlines challenging and adversely affect data quality (Menzi *et al.*, 2020 ▸; Chang *et al.*, 2022 ▸). For example, most cylindrical jets are too thick (typical diameters of 25 µm and larger) to enable soft X-ray transmission spectroscopy, where the transmission length of soft X-rays is only a few micrometres (Fondell *et al.*, 2017 ▸; Ekimova *et al.*, 2015 ▸). In addition, the thickness of a cylindrical jet varies strongly over the focal spot cross-section of the X-ray beam, making it difficult to determine the path length of the X-rays through the sample (Dupuy *et al.*, 2021 ▸). This hampers the precision of absorption measurements and can affect the intensity of the detected photoelectrons, introducing uncertainties in the X-ray photoelectron spectroscopy (XPS) analysis.

Several researchers have used liquid microjet sample introduction systems to measure the photoelectron angular distribution (PAD) from a variety of liquid samples. Studies of valence and core orbitals in liquid water (Ottosson *et al.*, 2010 ▸; Thürmer *et al.*, 2013 ▸; Nishitani *et al.*, 2017 ▸; Gozem *et al.*, 2020 ▸) have shown that the anisotropy is reduced in the condensed liquid phase compared with the gas phase. However, the angular distributions of electrons emitted from solutes have been less investigated. These measurements are complicated by the distribution of the solute near the surface. Photoelectron spectroscopy is a surface-sensitive method. Emitted photoelectrons undergo elastic and inelastic scattering before they exit the surface of the liquid, limiting both angular and energetic resolution as well as the escape depth of photoelectrons. Furthermore, the macroscopic curvature of the liquid surface complicates the interpretation of surface orientation effects. This makes it difficult to determine how the orientation of molecules at the interface influences their interaction with X-rays and their photoemission spectra (Dupuy *et al.*, 2022 ▸).

To address the aforementioned limitations, various approaches have been tested. Liquid flat jets have been generated by the collision of two Rayleigh jets (Rayleigh, 1879 ▸; Taylor, 1960 ▸). These jets have proven to be a promising alternative to cylindrical jets due to their near-planar flow (Ekimova *et al.*, 2015 ▸; Menzi *et al.*, 2020 ▸; Buttersack *et al.*, 2023 ▸). However, such traditional liquid flat jet nozzles require accurate alignment of the nozzle pair and relatively high liquid flow rates. High flow rates are problematic for scarce or valuable samples and for the maintenance of good vacuum conditions during operation. Kleine *et al.* (2019 ▸) recently reported static soft X-ray absorption measurements in transmission on the solvated compounds NH_2_CONH_2_, CaCl_2_, and NaNO_3_ using a flat jet generated via the collision of two cylindrical jets with an operational pressure in the liquid flat jet compartment below 1 mbar (maintained by the combination of a 1600 l s^−1^ turbopump and a liquid nitrogen cold trap).

Micro flat sheet jets have the potential to significantly improve the quality of X-ray scattering and spectroscopy experiments, especially for studies of aqueous samples (Barnard *et al.*, 2022 ▸; De Angelis *et al.*, 2024 ▸).

Steinke *et al.* (2016 ▸) developed a liquid jet setup for X-ray scattering experiments on complex liquids at free-electron laser sources (Hoffman *et al.*, 2022 ▸). The nozzle design described in that work can be further improved with 3D-printing technology. This method offers several benefits compared with conventional manufacturing methods, such as the ability to generate intricate geometries with high accuracy, low cost, and fast production. 3D-printing also allows for rapid prototyping for iterative design optimization, which ultimately leads to improved performance and efficiency of the nozzle system, as has been demonstrated by Konold *et al.* (2023 ▸).

To develop a compact flat sheet nozzle design and a universal sample delivery platform, we use 3D-printing technology. We can customize and create different nozzle designs that can be tailored to specific experimental needs and allow for complex flow behavior. The standardization of the flat sheet design increases the availability to new user groups. This facilitates the comparison and reproducibility of the experimental results by providing a consistent and reliable jet configuration.

Here, we show recent developments of 3D-printed nozzles intended for liquid jet spectroscopy that have been made possible via the availability of nozzle development and testing facilities at EuXFEL, Germany (Schulz *et al.*, 2019 ▸; Vakili *et al.*, 2022 ▸). In addition, we provide a first demonstration of the performance of one of these custom-designed nozzles in a new flat jet system that is currently under commissioning at MAX IV Laboratory in Lund, Sweden. The realization of such sheet jet arrangements for the MAX IV beamlines introduces a new injection method to analyze flat liquid surfaces, opening new possibilities for the spectroscopy community. The liquid flat jet platform reported here operates under vacuum, allowing soft X-ray absorption spectroscopy (XAS), XPS, and PAD measurements on aqueous solutions.

## 2. Material and methods

### 2.1. FlexPES beamline at MAX IV

The soft X-ray spectroscopy experiments described in this study were carried out at the photoemission endstation on branch B of FlexPES beamline (Preobrajenski *et al.*, 2023 ▸), on the 1.5 GeV ring at MAX IV Laboratory in Lund, Sweden. The beamline delivers photon energies in the range of 40–1500 eV with photon fluxes under normal working conditions ranging from 10^10^ to 10^12^ photons s^−1^. The storage ring operates in ‘top-up’ mode, with a nearly constant ring current of approximately 500 mA.

**Figure 1:**
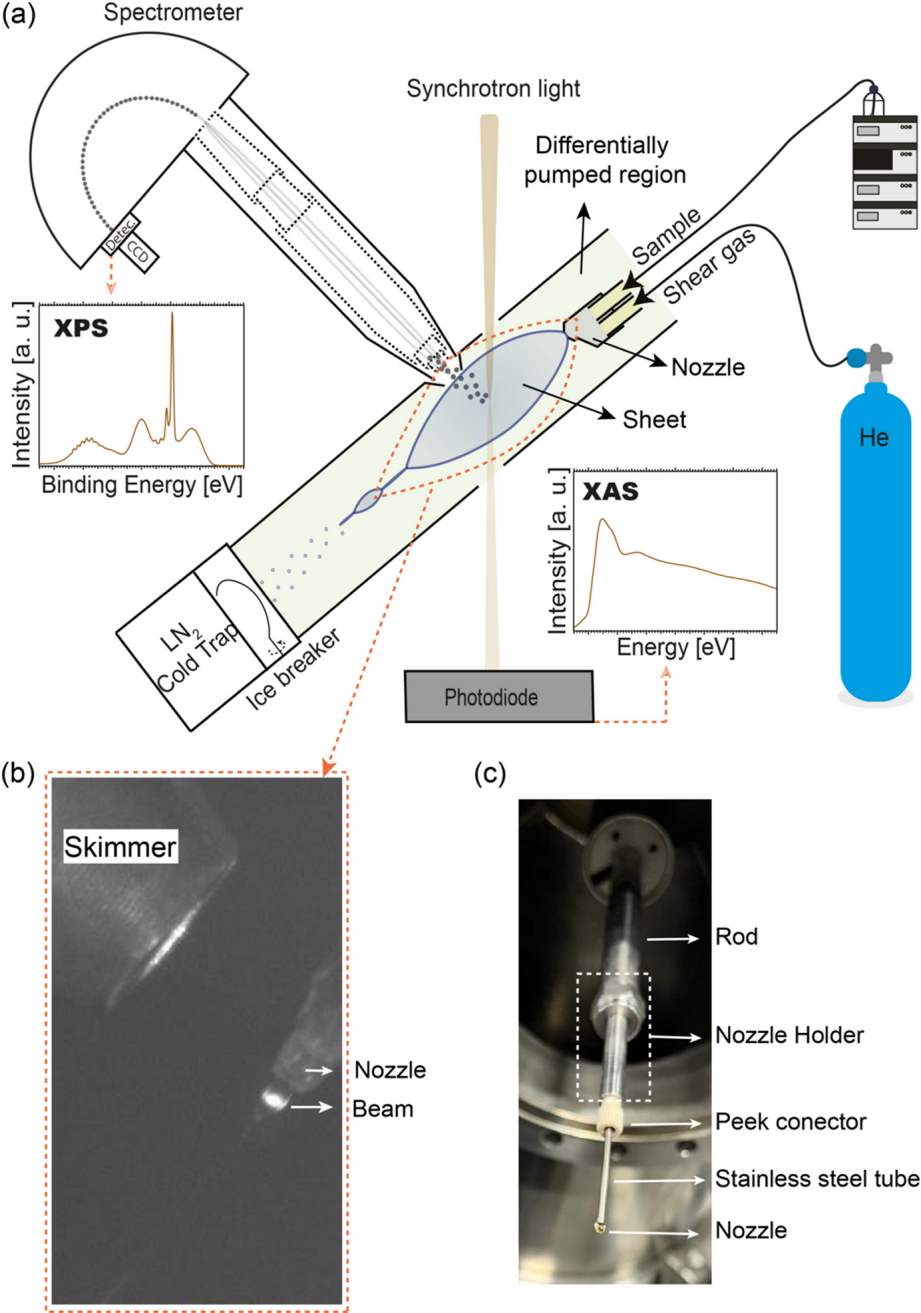
(*a*) Schematic of the liquid jet setup installed at the photoemission endstation at the FlexPES beamline. Note that the diagram is not drawn to scale. The nozzle position relative to the skimmer is not accurately represented for better visual clarity. (*b*) High-magnification image of the experimental arrangement including the 3D-printed nozzle position at a take-off angle near 0°, a skimmer with a 2 mm aperture diameter, and the white beam from the FlexPES beamline used to induce fluorescence of the saturated solution of Fluorescein, in the flat surface sample. (*c*) Standard nozzle holder and all key components used for mounting the 3D-printed nozzle assembled on the rod.

The photoemission endstation utilizes a Scienta R4000 hemispherical photoelectron analyzer. To allow transmission X-ray absorption measurements, an AXUV20A photodiode (Opto Diode Corporation) has been installed downstream of the endstation. The photodiode is located outside the differentially pumped interaction region of the endstation [Fig. 1 ▸(*a*)]. During an experiment, the liquid jet is directed into the interaction region perpendicular to the photon beam and the spectrometer lens axis. For these flat jet experiments, a new rod system was custom-designed to insert the injection devices into the photoemission endstation at FlexPES. The nozzle holder at the end of the rod is an adaptation of the one designed by Weierstall (2014 ▸); see Fig. 1 ▸(*c*).

To ensure a suitable vacuum in the interaction area, we installed a trap cooled by liquid nitrogen in the experimental chamber, mounted opposite the liquid jet nozzle [Fig. 1 ▸(*a*)]. This trap freezes the liquid beam and gaseous water molecules, effectively pumping the vapor out of the chamber. Aligning the liquid jet approximately 1 ± 0.1 mm from the skimmer cone opening of the electron spectrometer guarantees optimal performance. Electrons leaving the sample pass through the skimmer opening to reach the spectrometer.

The nozzle rod [Fig. 1 ▸(*c*)] is mounted on a rotary stage to vary the angle between the liquid sheet surface and the incoming radiation. This facilitates emission angle adjustment for the electrons traveling toward the spectrometer, which is installed perpendicular to the photon beam. Inside the rod, the sample is transported to the nozzle via 300 µm fused silica capillaries of approximately 3 m in length. Four-rod guides allow for the safe insertion of the rod through the manipulator, avoiding collisions of the nozzle tip with the support tube during installation. This eliminates the need for repeatedly mounting and dismantling the bulky liquid jet vacuum chamber for nozzle exchange. The interaction region located inside a differential pumping utilizes two HiPace 1500 turbomolecular vacuum pumps and was isolated from the spectrometer chamber via a skimmer cone with an opening diameter of 0.5 mm. The pressure inside the spectrometer chamber was approximately 2 × 10^−5^ mbar during experiments. The spectrometer requires low pressures for safe operation as well as for the production of high-quality data.

The spectrometer is positioned at an angle of approximately 90° to the polarization vector of the horizontally polarized light. The sample liquid is propelled through the capillary tubing and nozzle by an HPLC pump (Knauer Blueshadow 40P). The liquid flow rate is set to 0.4 ml min^−1^ (pressure ∼60–63 bar). The liquid lines incorporate a BIOTECH DEGASi *Plus* Semi-Prep degassing system to inhibit air bubble formation in the fluid stream. For the flat jet experiments described in this document, the flow rate of He was adjusted manually to approximately 9 mg min^−1^ using an AGA HiQ pressure regulator combined with a manual leak valve. The flow was monitored using a Bronkhorst F-111B mass flow meter.

Fig. 1 ▸(*b*) displays a high-magnification image of the experimental arrangement employed for XAS with a liquid sheet take-off angle of approximately 0°. The image emphasizes the most suitable position for the 3D-printed nozzle and the 2 mm-diameter skimmer. Moreover, the white beam from the FlexPES beamline, used to stimulate fluorescence in the solution, is also visible in Fig. 1 ▸. For that, a concentrated Fluorescein solution (C_20_H_12_O_5_, Thermo Scientific, with at least 90.0% purity) in ethanol (EMSURE ACS, ISO, Reag. Ph Eur, with at least 95.0% purity) has been prepared. Subsequently, we filtered the solution three times using Whatman Puradisc FP30 syringe filters with a pore size of 1.2 µm to eliminate any solid particles. Fluorescein produces visible fluorescence on the flat sheet when exposed to a white beam (produced over a wide range of energies) from the FlexPES beamline and hits the sheet through an exit slit measuring 100 µm in width. Based on the flat sheet angle between the nozzle and the camera (45°) and assuming an estimated scale using the skimmer base diameter of 2 mm, we estimated the beam spot size to be approximately ∼200 µm × 160 µm (V × H, FWHM).

For the experiments presented here, a 0.4 *M* (mol dm^−3^) aqueous solution of potassium formate was prepared by dissolving KHCOO (Thermo Scientific, ≥99.0% purity) in 0.2 mol% (∼0.1 *M*) ethanol (EMSURE ACS, ISO, Reag. Ph Eur, ≥ 95.0% purity) and de-ionized water (MilliQ, 18.2 MΩ cm). For the X-ray absorption measurements, a 1 *M* aqueous solution of ammonium nitrate was prepared by dissolving NH_4_NO_3_ (Sigma Aldrich, 99.0% purity) in de-ionized water. Before the measurements, each sample was filtered (Whatman Puradisc FP30 syringe filters, 1.2 µm) to remove solid particles. A photon energy of 360 eV and an electrical bias of −30 eV were used to move the gas phase signal out to the measurement windows. When an electric bias is applied during a liquid XPS experiment, the slope in the gas vacuum level and the gas signal broadening cause the gas phase position to shift. For the experiments discussed below, the beamline exit slit was opened to a width of 100 µm, which for the photon energies used in these experiments, corresponds to a photon energy resolution of 150 meV.

### 2.2. Nozzle fabrication and assembling at EuXFEL

The liquid sheet nozzles were printed with a Photonic Professional GT 3D-printer from Nanoscribe GmbH, which is based at EuXFEL (Schulz *et al.*, 2019 ▸). The printer utilizes a two-photon polymerization (2PP) mechanism to manufacture high-resolution structures at the sub-micrometre scale (Maruo *et al.*, 1997 ▸). In the 2PP process, a Ti-sapphire laser is directed through an objective lens into photo-resist resin (IP-S, Nanoscribe GmnH) to produce a final structure with a resolution of less than 100 nm.

To print complex 3D high-resolution structures, a droplet of UV-curaviscosity-resist IP-S resin was deposited onto a glass slide coated with indium tin oxide (ITO). This coating makes the glass slide highly conductive and transparent, but in our case, it is used to reflect the laser beam. Once the print is complete, several steps are required to clean and cure the photo-resist resin. Firstly, the glass slide was immersed in a propylene glycol monomethyl ether acetate (PGMEA; Sigma–Aldrich, ≥99.9% purity) solution for around 24 hours inside a beaker placed in a shaker to wash away the uncured material. Secondly, the nozzles were carefully transferred into 2-propanol (Millipore, C_3_H_8_O hypergrade for LC-MS LiChrosolv) to wash off any remaining PGMEA. Finally, after cleaning, the nozzles were placed in a fume hood under ambient conditions until completely dry.

The assembly of nozzles involves a multi-step process. Firstly, the nozzles were affixed onto a PDMS pad (Sylgard 184 silicone elastomer Kit from Dow Europe Gmbh) by means of Kapton adhesive tape. The Molex capillaries (ID 250 µm and OD 360 µm) were meticulously sliced using a ceramic blade to achieve a flat edge. The capillaries were inspected using microscopy to confirm they had been cut accurately. Two kinds of glue were used to connect the capillaries and 3D-printed nozzles: Devicon, 5 min Epoxy and Nordland Optical Adhesive 68 UV-glue. To apply the Epoxy, it was blended for 1 min and then placed on top of the capillaries and nozzle. A waiting time of 10 min was required to ensure the glue had set before proceeding to the next nozzle assembly. For the UV-glue, a drop of UV adhesive was applied, and it was cured using a Honle UV pencil for around 30 seconds.

The properties and performance of the liquid sheet were tested under atmospheric conditions with various liquid supply devices. For water, ethanol and their mixtures, an HPLC pump (Shimadzu Nexera 20AD-XR) was used, while glycerin and water solutions were tested using a mechanical syringe pump (Cetoni). The gas was provided using a Proportion AIR GP1 proportional gas regulator and the mass gas flow rate was tracked with a Bronkhorst F-111B flow meter. Optical images were captured using a Fastcam Mini AX200 (Photron) equipped with a 20× objective. The camera operated at a frame rate of 6400 fps and a shutter speed of 1/6400 s.

### 2.3. 3D-printed nozzle design

**Figure 2:**
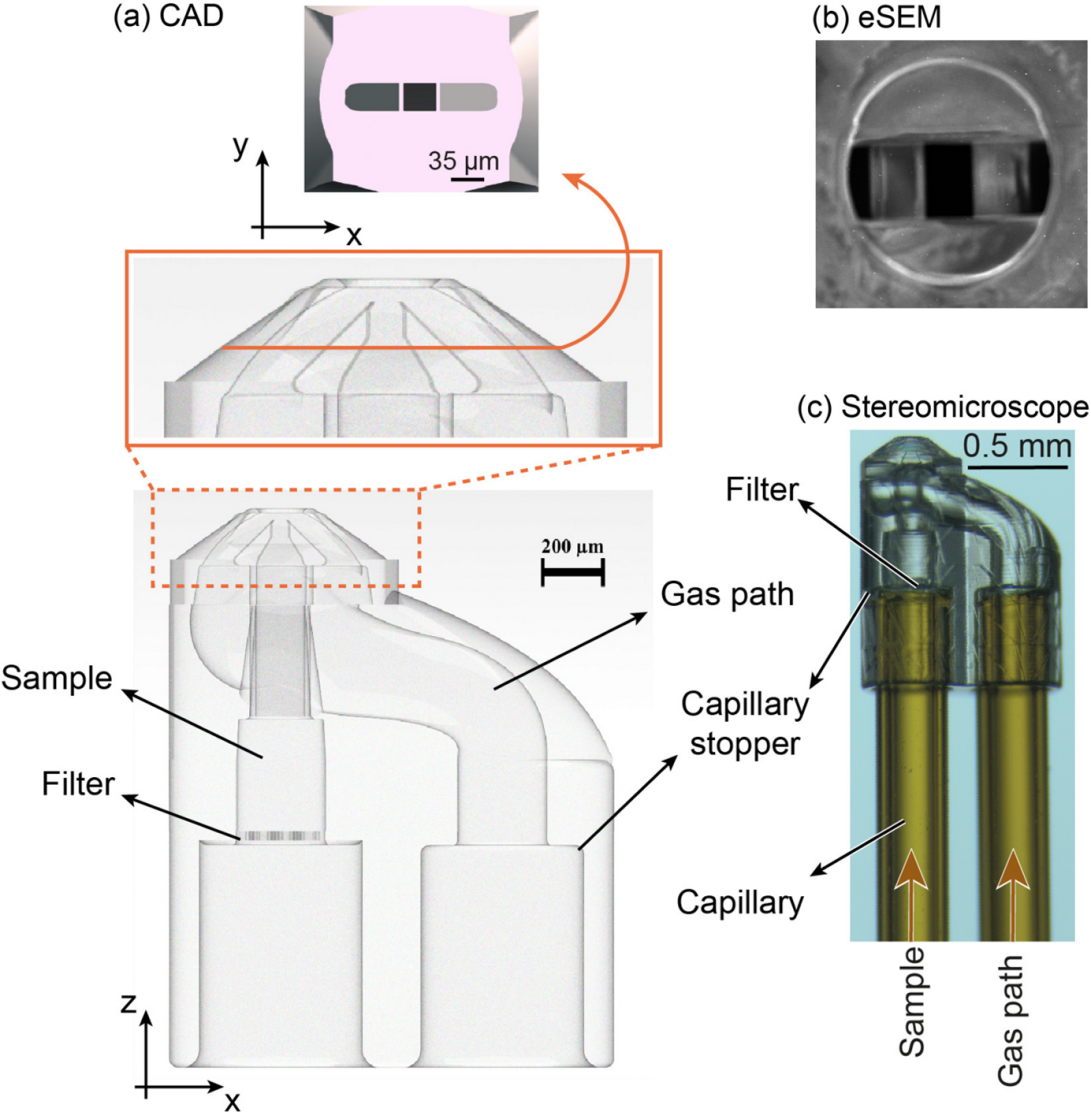
(*a*) 3D rendering of a CAD-model for a micro-sheet accelerated by gas. The 3D design of the nozzle presented at different angles is available in the supporting information. (*b*) Exit channels of the nozzle were imaged with an environmental scanning electron microscope. (*c*) 3D-printed nozzle with the two capillaries assembled before adding epoxy glue. The left capillary is used to insert the liquid sample, while the right one is connected to the He gas line. The image was captured with an Olympus SZX16 microscope and UC90 camera.

The nozzle design depicted in Fig. 2 ▸ utilizes photonic technology and 2PP to create a mixed 3D design capable of producing intricate structures with exceptional precision and detail. This design is divided into three sections: the receiving ports constitute the first section, whilst the intermediate transition region makes up the second. Both of these sections comprise more than 75% of the nozzle body. The end of the nozzle channels the sample flow through ducts with a quasi-rectangular cross-section, as illustrated in Figs. 2 ▸(*a*) and 2 ▸(*b*). The rectangular shape, in addition to gas compression, generates a thin and wide liquid sheet comparable to those formed by the commercially available glass nozzles from Micronit (Koralek *et al.*, 2018 ▸). The 3D-printed design also provides a steady jet and remarkable reproducibility, with 80% of printed devices achieving flawless performance.

For both gas and liquid channels [Figs. 2 ▸(*a*) and 2 ▸(*c*)], the ID is initially 365 µm. This is followed by a 540 µm-long section that decreases the ID to 200 µm, forming a capillary stopper. The final section is 330 µm long with a diameter of 100 µm for the sample channel. Additionally, a 40 µm pitch mesh filter was integrated into the sample channel to prevent suspended particles from clogging the nozzle exit (Knoška *et al.*, 2020 ▸).

In the middle section, the gas pathway divides into two channels that converge at a 45° angle to cut the liquid and enhance the sheet surface. In the final collision region between the gas and liquid, both the liquid and the gas channels are restricted to a 35 µm × 35 µm cross-section of the duct. In this arrangement, the sample exit is 15 µm from the nozzle exit. For a better understanding of the design, a 3D model of the nozzle illustrating different angles of the design is available in the supporting information, together with a .stl file containing the 3D model and the dimensions of the nozzle.

**Figure 3:**
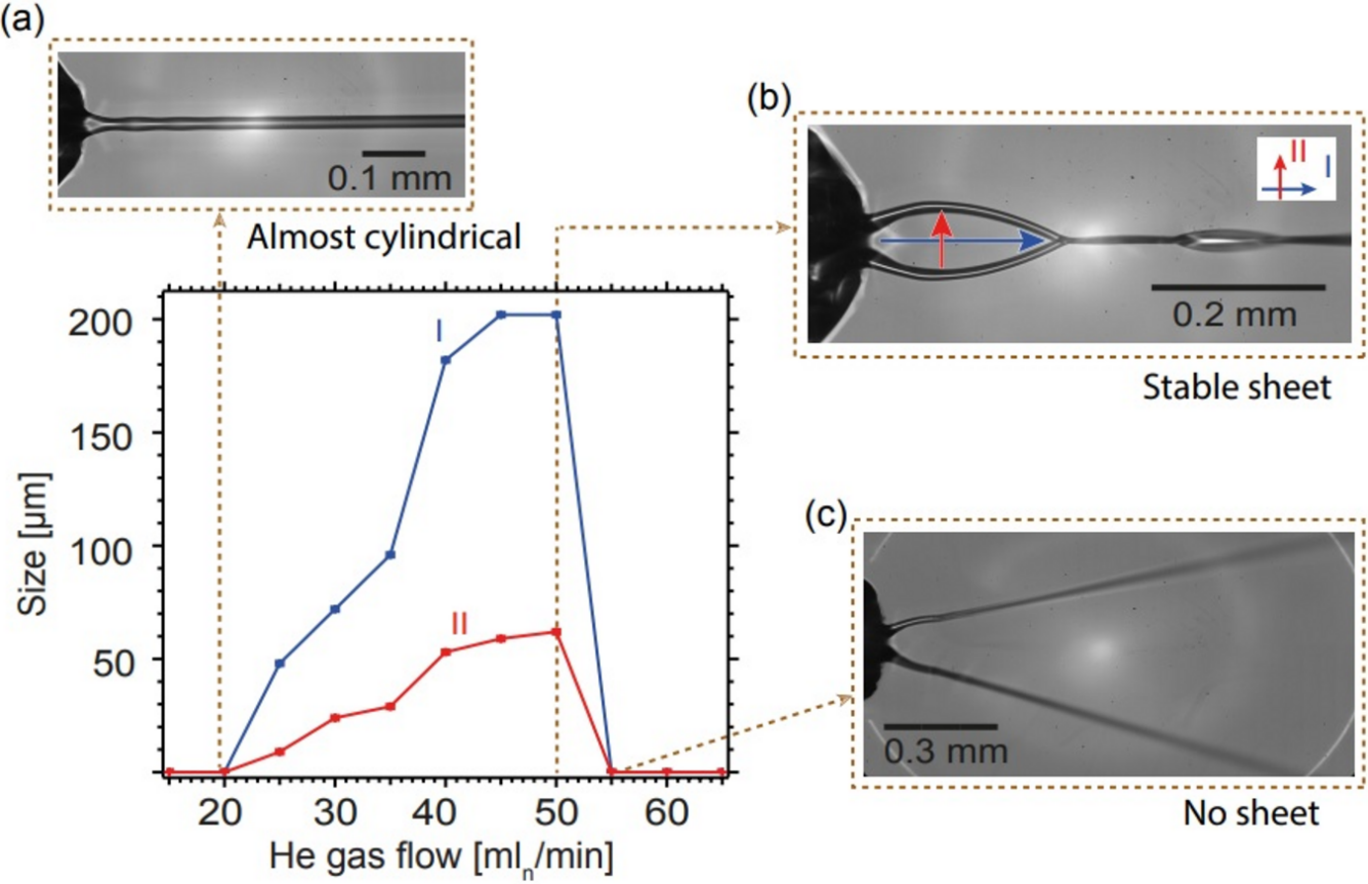
Illustration of the flat sheet jet size (length I and width II) for a varying He gas and a fixed water flow rate of 0.25 ml min^−1^ under atmospheric conditions. Panels (*a*) and (*c*) Instances of low and high He flow rates, respectively, forming an almost round jet and a spray as a result of a broken flat sheet jet, while panel (*b*) shows the formation of the largest and most stable flat sheet jet.

Fig. 3 ▸ illustrates the behavior of the nozzle at a constant water flow rate of 0.25 ml min^−1^ and varying gas flow rates. Due to the He gas flow, multiple flat liquid sheets are formed in the sample jet. At gas flow rates below approximately 20 ml min^−1^, the jet is not significantly affected by gas compression.

The first sheet formed near the nozzle collapses, and a second, smaller, perpendicular sheet is formed. This pattern is repeated, resulting in the jet containing a series of successive sheets of decreasing size, as shown in Fig. 3 ▸(*b*). Figs. 3 ▸(*a*) and 3 ▸(*c*) depict instances where the He flow rates were too low and too high, respectively, to form a stable liquid sheet. To achieve the largest stable flat sheet (length 200 µm × width 50 µm), such as the one shown in Fig. 3 ▸(*b*), it is necessary to maintain the gas flow within a specific range (50 ml min^−1^). When the gas flow is insufficient [Fig. 3 ▸(*a*)], the liquid jet takes on an almost cylindrical shape. Conversely, excessive gas flow [Fig. 3 ▸(*c*)] leads to sheet breakage. In this case, the sheet breaks up at the intersection of the first and second sheets, causing the liquid rims to spread in an unstable manner that prevents sheet formation and enters a spraying mode.

Furthermore, the thickness (in the middle of the flat sheet) of a water sheet was measured using the white-light interferometer system. The results indicate that a thickness of ∼2–4 µm – using ∼0.25 ml min^−1^ and ∼50 ml_n_ min^−1^ liquid and gas flows, respectively – could be achieved.

## 3. Results and discussion

The high stability and optical flatness of the liquid sheet jets make them an ideal choice for beamline applications. These properties also provide valuable insights into the optimal design for measurements at the FlexPES beamline. We used the flat sheets to investigate the XPS of potassium formate in ethanol. Ethanol is a volatile organic compound that can easily adsorb on the liquid–vapor interface, while potassium formate is an ionic salt that dissolves in water and forms potassium and formate ions.

The detection of electrons ejected from a surface depends on the take-off angle of the detected photoelectrons from the surface. A shallow take-off angle results in a smaller mean escape depth of the electrons than a large take-off angle. Considering the short inelastic mean free path of the electrons for the kinetic energies used here (Thürmer *et al.*, 2013 ▸), at a take-off angle of ∼10°, the photoelectrons are emitted from a very thin layer near the surface, with an estimated escape depth of several nanometres to tens of nanometres. Therefore, the measurement is more sensitive to molecular orientation and interfacial structure.

**Figure 4:**
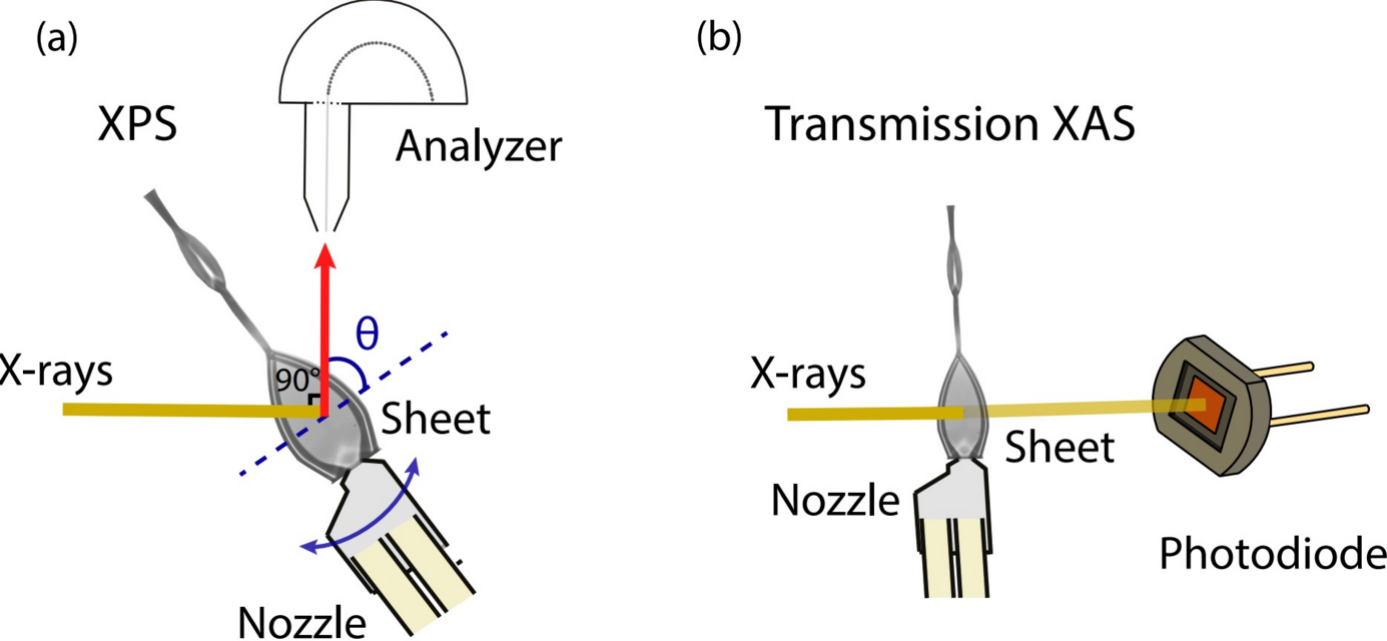
Diagram of the geometry for (*a*) XPS and PAD, where the sample take-off angle can be varied by tilting the sample relative to the detector; and (*b*) soft X-ray transmission spectroscopy on a flat surface.

To determine the take-off angle, we set up a camera in line with the spectrometer lens axis to establish at which angle the first liquid sheet was narrowest when rotating with the rotary stage. That angle was then defined as the zero take-off angle, which can be seen in Fig. 4 ▸(*a*). We anticipate that this method results in a 5° uncertainty in the take-off angle.

In order to investigate the surface composition through a flat jet, we changed the take-off angle of electrons from the surface of the jet towards the spectrometer [see Fig. 4 ▸(*a*)].

Ethanol is a known surfactant in the sense that it lowers the surface tension of aqueous solutions (Vazquez *et al.*, 1995 ▸), and it is therefore expected to adsorb at the liquid–vapor interface. This has earlier been shown to be the case in photoelectron spectroscopy measurements using cylindrical liquid jets (Marinho *et al.*, 2017 ▸). In contrast, both potassium and formate ions are known to increase the surface tensions of aqueous solutions (Marcus, 2010 ▸) and are thus expected to avoid the surface, in accordance with electrostatic theory. A limitation of using the flat sheets for these types of measurements is the thicker rim formed at the edges of the sheet, which makes it difficult to investigate very small take-off angles (Menzi *et al.*, 2020 ▸; Koralek *et al.*, 2018 ▸). As a result, the measurements were limited to angles of ∼10° or more, as we assumed in agreement with the size of the flat sheet edges that the thicker rim would not influence the data beyond this point.

**Figure 5:**
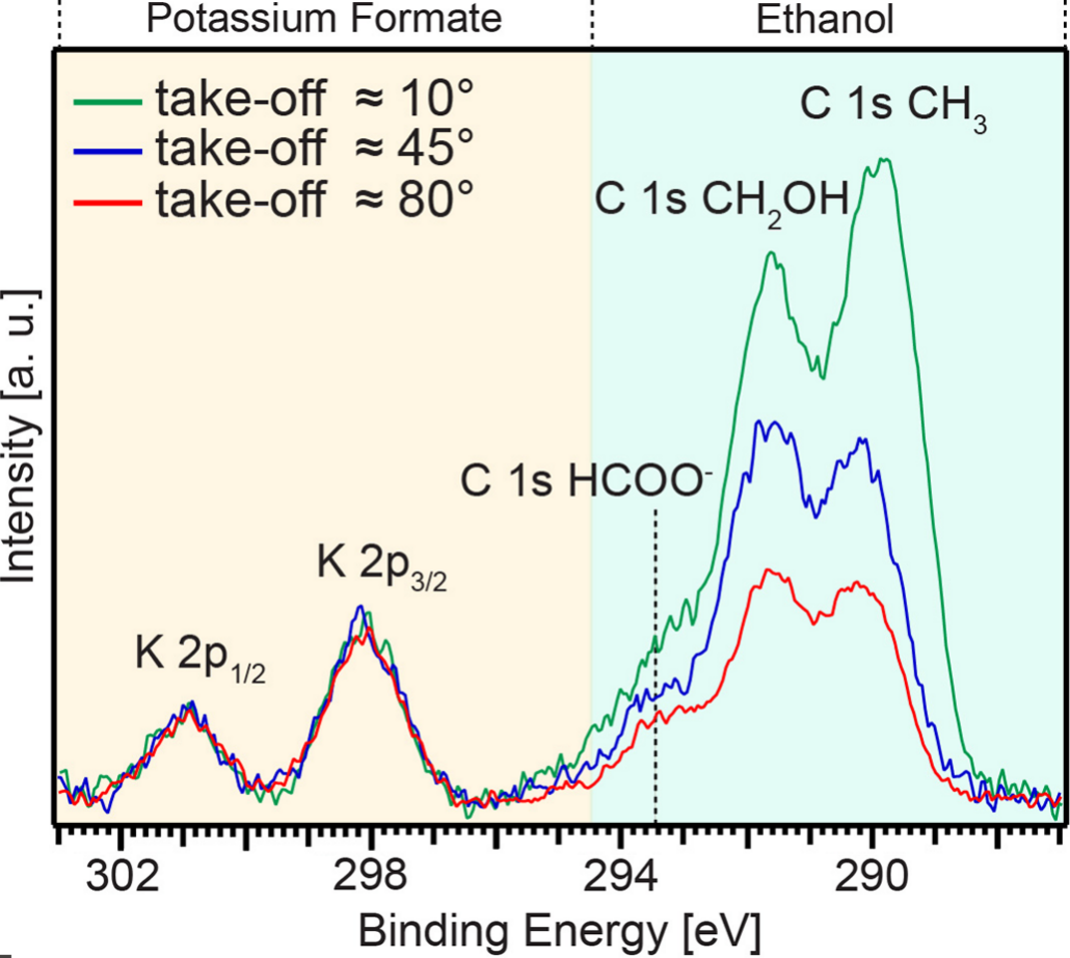
C 1*s* photoelectron spectra from an aqueous solution of 0.4 *M* potassium formate (KHCOO) in 0.2 mol% (0.1 *M*) ethanol. The spectra were recorded at a photon energy of 360 eV and with the lens axis at 54.7° with respect to the horizontally polarized radiation. The take-off angles were approximately 10° (green line), 45° (blue line), and 80° (red line). The spectra were normalized to K 2*p*.

Fig. 5 ▸ shows the photoelectron spectra recorded following C 1*s* core-level excitation from an aqueous solution of 0.4 *M* potassium formate (KHCOO) in 0.2 mol% (0.1 *M*) ethanol. The spectra were measured at take-off angles of 10, 45, and 80°. The photon energy was set to 360 eV, and the analyzer was operated at an angle θ = 54.7° with respect to the polarization vector of the horizontally polarized radiation. The peaks located at ∼301 eV, ∼298.2 eV, and ∼293.5 eV are attributed to K 2*p*_1/2_, K 2*p*_3/2_, and C 1*s* of HCOO^−^, respectively, from potassium formate, relying on energies reported in previous studies (Ottosson *et al.*, 2011 ▸; Brown *et al.*, 2012 ▸). For ethanol, the C 1 s peaks for CH_2_OH and CH_3_ are located at ∼291.6 eV and ∼290 eV, respectively (Marinho *et al.*, 2017 ▸). The spectra have been normalized to the K 2*p*_3/2_ area, and a polynomial background was subtracted from all spectra.

The normalized intensities of the ethanol component reveal a notable contrast variation among the measured take-off angles, clearly indicating the higher concentration of ethanol at the surface. This attests to the effectiveness of 3D-printed flat sheet nozzles in producing a capacious, steady and uniform sheet.

### 3.1. Soft X-ray transmission spectroscopy of liquid solutions

Soft XAS is a method that involves exciting a core electron to an unoccupied state to study the electronic and geometric structure around specific atoms in liquid samples. Although transmission-type liquid flow cells have been developed to reduce the absorbance of liquid samples by controlling the liquid thickness, liquid flat jet systems have several advantages over them. Liquid flat jet systems produce stable liquid sheets with controlled thickness and size, which allows for better control over the sample environment. Additionally, the liquid flat jet operates with full function under vacuum conditions, allowing soft X-ray spectroscopy of aqueous solutions in transmission mode. This makes it possible to investigate the local structures of several types of liquid solutions using transmission-mode XAS techniques. The measurement geometry is schematically shown in Fig. 4 ▸(*b*).

**Figure 6:**
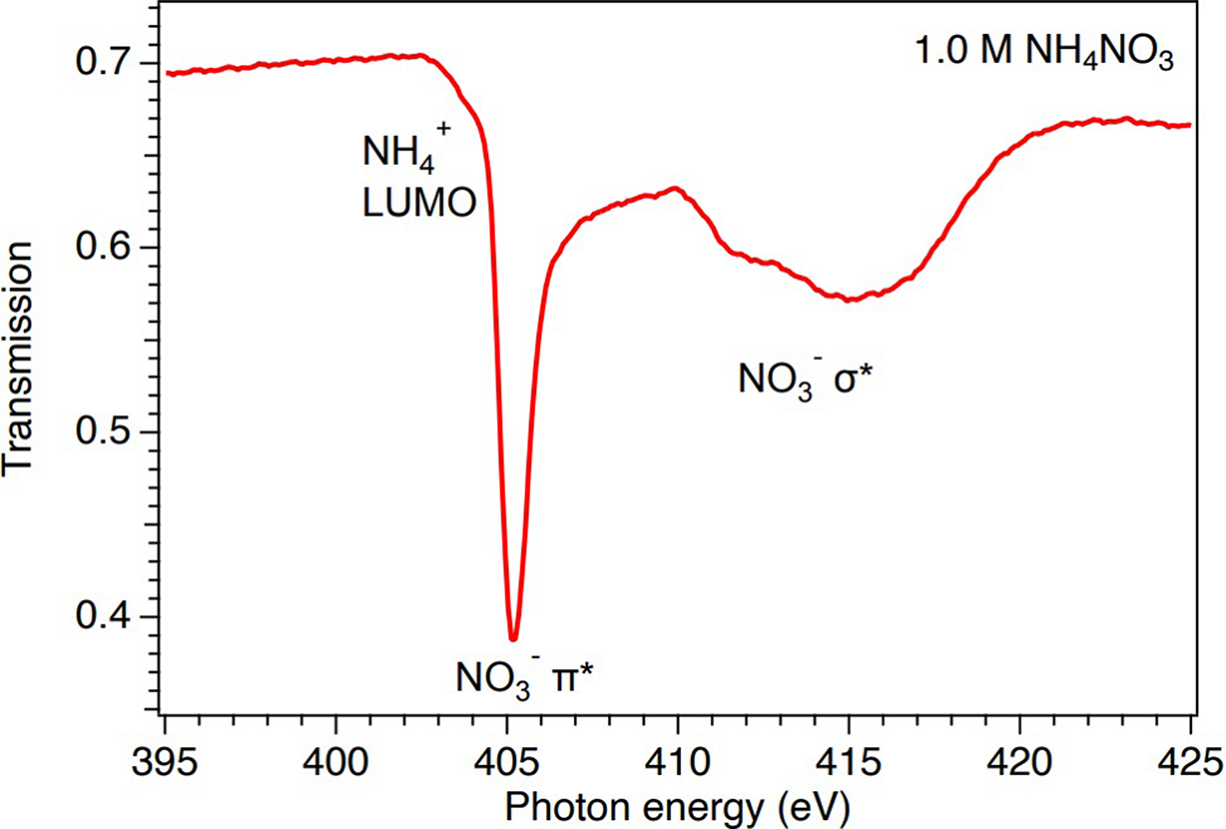
N 1*s* spectrum obtained by soft X-ray transmission measurements of an aqueous solution containing 1.0 *M* NH_4_NO_3_ using a 3D-printed nozzle.

As an example, in Fig. 6 ▸, we show the N *K*-edge X-ray absorption spectrum measured in transmission mode from an aqueous solution of 1.0 *M* ammonium nitrate (NH_4_NO_3_) using a 3D-printed nozzle. The spectrum has been normalized to the photon flux measured by the photodiode with the liquid jet lowered out of the path of the photon beam. The bulk solvation of aqueous ammonium (Ekimova *et al.*, 2017 ▸, 2018 ▸; Carter-Fenk & Head-Gordon, 2022 ▸) and nitrate (Smith *et al.*, 2015 ▸) ions have been investigated earlier using experimental N 1*s* near-edge XAS and theoretical modeling. Briefly, the prominent peak at 405 eV has been attributed to the π* resonance in NO_3_^−^ and the broad structures around 412–415 eV to σ* resonances in NO_3_^−^ (Smith *et al.*, 2015 ▸). The spectrum is less structured for aqueous NH_4_^+^ and most of it overlaps with the more prominent NO_3_^−^ signal, but the shoulder observed around 404 eV is the pre-edge peak of NH_4_^+^ (Ekimova *et al.*, 2017 ▸; Carter-Fenk & Head-Gordon, 2022 ▸). By comparing the experimental transmission just before the absorption features (∼0.7 at 400 eV) to that derived from tabulated atomic data (Henke *et al.*, 1993 ▸), we estimate an average thickness of ∼1.7 µm of the illuminated area of the flat jet leaf. This thickness value has been confirmed by interferometer studies. More details on the thickness determination will be presented in a subsequent paper. In all, the data presented in Fig. 6 ▸ demonstrate the feasibility of transmission-mode XAS measurements in liquids in the soft X-ray regime using the 3D-printed nozzles described here.

## 4. Conclusions

The high stability and optical flatness of liquid sheet jets are crucial properties for soft X-ray experiments under vacuum, as they allow the study of a wider range of systems in their natural liquid or solution phases. The 3D-printed flat sheet nozzle is a promising alternative to cylindrical microjets for soft X-ray liquid spectroscopy due to its properties. We have developed a 3D-printed flat sheet nozzle and liquid jet environment to implement it for the first time on the FlexPES beamline at MAX IV. Our 3D-printed nozzle design produces planar jets of the order of micrometres in thickness, large enough to cover the beam spot in focus in FlexPES, and very stable at low sample and gas flow rates. This allows acceptable vacuum conditions in the main chamber of the FlexPES beamline.

We studied C 1*s* photoelectrons for an aqueous solution containing 0.2 mol% (0.1 *M*) ethanol and 0.4 *M* potassium formate. For the first time on the FlexPES beamline, we used a flat sheet jet produced by a 3D-printed nozzle and a new jetting platform for these measurements. The spectra were recorded at 54.7° relative to the horizontal polarization of the synchrotron radiation, with a photon energy of 360 eV and take-off angles of 10°, 45°, and 80°.

The normalized intensities for the ethanol constituent display a markedly high contrast between the measured take-off angles, demonstrating the effectiveness of the developed injection system in generating large, stable, and uniform sheets, which leads to high surface yield.

This study reveals the potential for utilizing a 3D-printed flat sheet nozzle in soft X-ray spectroscopy of liquid samples. The 3D flat jet generates a thin, stable, and optically flat liquid sheet that increases the surface sensitivity and signal quality of the photoelectron spectra compared with a cylindrical nozzle. Furthermore, it provides additional flexibility and versatility in design and fabrication. The excellent performance of the flat jet produced in a vacuum helps us to conduct XPS and PAD measurements as well as XAS experiments more accurately and precisely. Furthermore, we prove the feasibility of transmission-mode XAS measurements in liquids in the soft X-ray range, utilizing 3D-printed nozzles.

## Supplementary Material

3D model of the nozzle, illustrating different angles of the nozzle design for better clarity. DOI: 10.1107/S1600577524006611/ye5047sup1.pdf

For a better understanding of the nozzle design, a 3D model of the design of the flat sheet nozzle can be found in the .stl file. DOI: 10.1107/S1600577524006611/ye5047sup2.bin
