# Supplementary material for: Development of a flat jet delivery system for soft X-ray spectroscopy at MAX IV
Source: J Synchrotron Radiat. 2024 Aug 22;31(Pt 5):1285–92. doi: 10.1107/S1600577524006611 (PMC11371042; doi:10.1107/S1600577524006611)
Supplement: Supplementary file 1 [file s-31-01285-sup1.pdf]

JOURNAL OF SYNCHROTRON RADIATION

Volume 31 (2024)

Supporting information for article:

Development of a flat jet delivery system for soft X-ray spectroscopy at MAX IV

Tamires Gallo, Luigi Adriano, Michael Heymann, Agnieszka Wrona, Noelle Walsh, Gunnar Öhrwall, Flavia Callefo, Slawomir Skruszewicz, Mahesh Namboodiri, Ricardo Marinho, Joachim Schulz and Joana Valerio

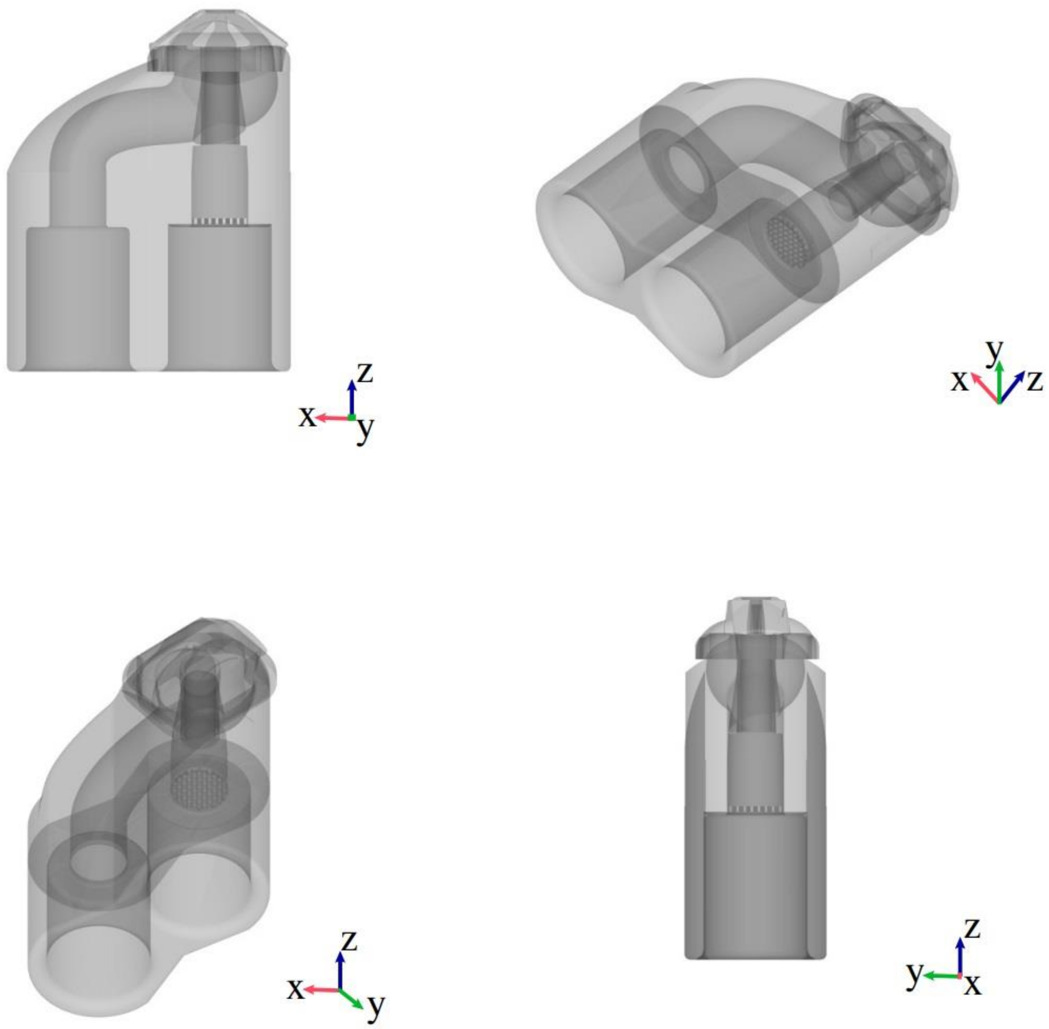

Figure S1 3D model of the nozzle, illustrating different angles of the nozzle design for better clarity.
